# The Overtreatment Trap: Navigating Dopamine Dysregulation Syndrome in Parkinson's Disease

**DOI:** 10.7759/cureus.70500

**Published:** 2024-09-30

**Authors:** Vibhootee Sant Bakshsingh, Ruford Sequeira

**Affiliations:** 1 Internal Medicine, Royal Gwent Hospital, Newport, GBR

**Keywords:** dopamine dysregulation syndrome, dopamine replacement therapy, impulse control and behavioural disorders, levodopa-induced dyskinesia (lid), parkinson's disease, prescription drug addiction, punding

## Abstract

Dopamine dysregulation syndrome (DDS) is a rare but significant complication of Parkinson's disease (PD), affecting approximately 3-4% of patients on long-term dopamine replacement therapy (DRT). It is characterized by an addictive pattern of DRT use that exceeds the necessary dosages for managing motor symptoms. Patients may engage in self-medication, escalating their DRT doses beyond prescribed limits, and strongly resist attempts to reduce medication. This syndrome often leads to impulsive behaviors, severe dyskinesia, and notable disruptions in social and occupational functioning. DDS is associated with a range of neuropsychiatric symptoms, including punding behaviors, hallucinations, and delusions. The management of DDS presents significant challenges, requiring a delicate balance between adequate symptom control and preventing medication overuse. We present a case of a 68-year-old woman with DDS, highlighting her symptoms and the tailored management strategies we employed to address this challenging condition.

## Introduction

Parkinson's disease (PD) is a chronic, progressive neurodegenerative disorder characterized primarily by motor symptoms such as resting tremor, rigidity, and bradykinesia. These symptoms result from the degeneration of dopaminergic neurons in the substantia nigra pars compacta [1]. As the disease progresses, patients often experience a range of non-motor symptoms, including cognitive impairment, mood disorders, sleep disturbances, and autonomic dysfunction [1-2].

The cornerstone of PD management is dopamine replacement therapy (DRT) [2]. However, prolonged exposure to DRT, combined with the underlying dopaminergic system degeneration, can lead to impulse control and behavioral disorders (ICBD) in approximately 14% of patients [3]. A particularly challenging subset of ICBD is the dopamine dysregulation syndrome (DDS), affecting 3-4% of PD patients in tertiary care settings [4].

DDS is characterized by the compulsive overuse of dopaminergic medications, often exceeding the necessary doses to manage motor symptoms. This disorder stems from chronic, excessive dopaminergic drug intake, particularly high doses of levodopa. The overuse leads to pulsatile stimulation of dopamine receptors, which sensitizes the associative-limbic dopaminergic system. As a result, the mesolimbic dopamine system becomes overstimulated, amplifying the rewarding and motivational effects of these medications. This overstimulation drives patients to compulsively seek and consume these drugs, even when their motor symptoms are adequately controlled [5]. Recent post-mortem studies of PD patients with impulse control behaviors, including those with DDS, have shown reduced α-synuclein deposition and lower dopamine D3 receptor levels in the nucleus accumbens. These findings are believed to result in excessive stimulation of the ventral striatum, which may further reinforce addictive behaviors and impulsive-compulsive tendencies in these patients [6].

Managing DDS poses a significant challenge in PD care, requiring a careful balance between controlling motor symptoms and preventing medication overuse. This complex condition necessitates a multidisciplinary approach involving Parkinson's specialists, psychiatrists, and other healthcare professionals to address both the motor and behavioral aspects effectively [1].

We present a case of a 66-year-old woman with PD who developed DDS, highlighting the complex interplay between disease progression, medication management, and behavioral changes. Our case illustrates the clinical manifestations of DDS and explores the multifaceted approach required for its management.

## Case presentation

This case report chronicles the journey of a woman diagnosed with idiopathic PD at age 66 in 2007, though her symptoms had begun at 62. Initially treated with Sinemet, her medication regimen was gradually increased and expanded to include pramipexole due to worsening bradykinesia and tremors. At this point, she was able to mobilize independently. Over the next three years, her mobility declined, partly due to severe osteoarthritis, which led to bilateral total knee replacements during this period. However, her mobility continued to deteriorate, and she attributed her ongoing pain to her PD progression. Her medications were gradually further increased over the next four years. Notably, during these four years, the patient had eight consultations, of which five were telephonic conversations, and all medication increases were done during these telephonic consultations.

By 2014, the patient, now weighing only 43.5 kg, was on an estimated levodopa equivalent dose (LED) of 2,308 mg [7]. Her medication regimen was as follows: Stalevo (levodopa, carbidopa, and entacapone) 150/37.5/200 mg, one tablet five times per day; Sinemet CR (co-careldopa) 50/200 mg, one tablet twice per day; Sinemet plus (co-careldopa) 125 mg (50/100 mg), two tablets twice per day; rasagiline 1 mg, one tablet once daily; Madopar (co-beneldopa) dispersible 62.5 mg (12.5/50 mg), one tablet four times per day; pramipexole (Mirapexin) prolonged-release 2.1 mg, one tablet once daily; and amantadine 100 mg, one tablet once daily

Despite this intensive medication regimen, she continued to struggle with her PD symptoms, particularly freezing of gait, and reported significant pain, especially at night. She also exhibited marked dyskinesia, which may have contributed to her discomfort. Additionally, she developed dystonia, leading to nighttime neck pain. Her husband, who accompanied her to appointments, mentioned that she had been engaging in obsessive behaviors such as gardening, painting furniture, and tidying over the past few months.

Concerns regarding her symptoms and high medication doses prompted the Parkinson's specialist to consider DDS, leading to a proposed plan for the gradual tapering of her medications. The initial plan involved reducing Madopar 62.5 mg from four times daily to twice daily, discontinuing rasagiline, and lowering Sinemet plus from 250 mg twice daily to 187.5 mg twice daily. This suggestion was met with strong resistance from both the patient and her husband, who argued vehemently for maintaining current doses, fearing a resurgence of symptoms if reduced. The patient, torn between her dependency on the medications and her desire not to feel like "a drug addict," became tearful and anxious. She insisted that her high medication needs were a result of the disease, not personal fault. After extensive explanation and reassurance, she reluctantly agreed to try the reduced regimen.

However, the subsequent consultation revealed a concerning picture. The patient had not only failed to decrease her medications as agreed but had increased her intake, using Madopar as an ad-hoc pain relief medication. She also began experiencing tactile hallucinations, describing sensations of insects crawling over her skin. The patient firmly rejected further medication reductions, arguing that the medical team's recommendations were causing her increased stress and anxiety, with her husband fully backing her position. Over the following months, her condition worsened: her dyskinesia intensified, and her mobility declined to the point where she required mobility aids.

For the next four years, despite efforts to reduce her prescribed medications, the patient continued to obtain and take medications beyond her prescriptions. Her dyskinesia worsened, and she developed a range of concerning symptoms. Her compulsive behaviors, such as constant tidying and excessive gardening, were progressively worsening. These behaviors, which consumed much of her time and energy, were starting to interfere with her daily life and overall well-being. She also exhibited paranoia, particularly jealousy when her husband interacted with carers and increased irritability that manifested as verbal abuse. Her hallucinations expanded to include both auditory experiences, like hearing children singing, and visual ones, such as seeing people and insects on the roofs. Additionally, she developed delusions, including beliefs about passing worms in her stool. A psychiatric evaluation confirmed that she was experiencing hallucinations and delusional thoughts and was advised for hospital admission to facilitate medication reduction and improve her symptoms, but she declined the offer.

A turning point came when she was admitted to the hospital in 2018 by the gastroenterology team for investigation of her reported stool issues. While these investigations yielded no significant findings, the admission provided an opportunity to review and adjust her medication regimen. Her new regimen was adjusted to a total LED of 2,198 mg while still effectively managing her PD symptoms.

Over the following two years, she unfortunately had two more hospital admissions for a rectal prolapse and a femur fracture. These hospitalizations, while challenging, provided crucial opportunities for further medication adjustments. Her Parkinson's medication regimen was reduced to an estimated LED of 1,048.5 mg, leading to significant improvements in her condition. Hallucinations, though not entirely gone, were notably reduced, with only occasional reports of seeing small insects in the room. Her delusions were fully resolved. Although some obsessive behaviors, like rearranging papers and magazines, persisted, they were less intense and disruptive than before. Her emotional state also improved markedly, with a more stable temperament and reduced emotional lability. Most impressively, despite the significantly lower medication dose, her motor symptoms remained well-controlled, and there was a significant reduction in her dyskinetic movements. These positive changes collectively painted a picture of significant overall improvement in her quality of life and management of her PD symptoms.

Perhaps one of the most striking changes was in the attitude of her husband. Initially resistant to any medication reduction and supportive of his wife's requests for more medication, he now embraced the positive changes. In fact, he even suggested further reducing her medication, proposing to eliminate the 5:00 PM dose of Sinemet 62.5 mg, noting that she already had Stalevo at 4:00 PM and another Sinemet dose at 7:00 PM. This adjustment would bring her LED down to approximately 998.5 mg [7]. Her revised medication regimen would be as follows: Stalevo (levodopa, carbidopa, and entacapone) 150/37.5/200 mg, one tablet three times per day; Madopar (co-beneldopa) dispersible 62.5 mg (12.5/50 mg), one tablet once daily; Sinemet plus (co-careldopa) 125 mg (25/100 mg), one tablet once daily; Sinemet (co-careldopa) 62.5 mg (12.5/50 mg), one tablet three times per day; and amantadine 100 mg, one tablet once daily.

The patient herself experienced a significant shift in her outlook. Her previously overwhelming anxieties about reducing her medication gave way to a profound understanding of its crucial role in her overall well-being.

## Discussion

This case study demonstrates the complexities of managing DDS in PD, offering valuable insights into the challenges faced by both patients and healthcare providers while emphasizing the potential for successful outcomes through consistent and dedicated care.

Initially described by Giovannoni et al. as hedonistic homeostatic dysregulation syndrome, DDS is characterized as a neuropsychological disorder associated with substance misuse and addiction [2,8]. Giovannoni et al.'s study provides a comprehensive overview of the syndrome's progression and manifestations [2]. In the early stages, patients frequently engage in self-medication, leading to rapid and excessive increases in their dopaminergic replacement therapy (DRT) doses, often far beyond what is necessary to manage motor symptoms. Consequently, patients end up taking larger and more frequent doses, significantly increasing their total daily intake of levodopa. Patients typically resist efforts to reduce their DRT, developing tactics to hoard medications and circumvent physicians' attempts to limit their usage. By the time the misuse of DRT and the associated behavioral disorder become clinically apparent, many patients have developed severe, albeit tolerated, drug-induced dyskinesias that do not deter them from further increasing their DRT doses. Patients often neglect prescribed dosing regimens and self-medicating based on somatic cues that may not correlate with PD symptoms. Their perception of the "on" state, which is when they experience temporary relief from their PD symptoms, becomes distorted, with patients reporting a sense of being "on" only when experiencing marked dyskinesia. Their study also showed that as DRT levels rise, patients may experience hypomanic behaviors that can progress to manic psychosis, characterized by psychomotor agitation, increased excitability, elevated mood or euphoria, irritability, low frustration tolerance, disorganized thinking, and poor judgment. Paranoia is also prevalent, often directed at hospital staff, caregivers, and family members, complicating efforts to manage DRT. Rapid mood fluctuations and aggressive behavior may occur as well [2].

These patterns were also observed in our patient, whose increasing reliance on dopaminergic medications, resistance to dose reductions, and distorted perception of the "on" state closely align with the classical DDS presentations described in the literature. Her use of Madopar as a "pain relief" medication exemplifies how patients with DDS may self-medicate based on somatic cues unrelated to their PD symptoms. Additionally, her experiences of paranoia, delusions, and hallucinations reflect the complex interplay of psychiatric symptoms documented in DDS cases.

DDS is also linked to punding, characterized by stereotyped behaviors involving complex, excessive, and non-goal-oriented activities [9]. Fasano and Petrovic highlighted gender-specific patterns of repetitive behaviors in individuals with punding [10]. Men often engage in activities like obsessively tinkering with technical objects such as radios, clocks, watches, or car engines, meticulously analyzing, organizing, and cataloging parts, but seldom reassembling them. Women, on the other hand, are more likely to engage in behaviors such as continuously sorting through their handbags, persistently tidying, brushing their hair, or polishing their nails. Despite recognizing that their actions are excessive and purposeless, individuals with punding continue these behaviors, even to the point of self-harm. They noted that this compulsive behavior is most commonly linked to DRT in PD patients and is similarly observed in individuals using cocaine and amphetamines [10]. The development of punding behavior in this patient, manifested as excessive gardening, tidying, and repetitive furniture painting, is interestingly reflected in this case as well.

As the response to DRT varies among individuals, there is no established dose cut-off for DDS. However, physicians should be vigilant when patients exceed 2,000 mg of levodopa or take more than eight rescue doses (including apomorphine injections) daily [2]. Our patient was taking approximately 2.3 g of levodopa per day, which significantly surpasses this limit.

The reasons some individuals with PD are more susceptible to DDS remain unclear. However, several key risk factors have been identified, including male gender, younger age at disease onset, higher novelty-seeking personality traits, depressive symptoms, and a personal or family history of substance misuse. Furthermore, impulsive personality traits, genetic traits related to dopamine receptors, and cognitive deficits that impair decision-making can also contribute to the risk of developing DDS [11-13].

Managing DDS is complex. While it is a recognized condition affecting individuals with PD, its management largely relies on physicians' personal experience due to the absence of clear management guidelines. DDS is difficult to characterize solely as an addiction, particularly as patients often require increasing doses of dopamine replacement therapy due to the progressive loss of their dopaminergic neurons [8]. The National Institute for Health and Care Excellence (NICE) guidelines advise that patients with impulse control behaviors should be managed by healthcare professionals specializing in PD before making changes to their dopaminergic therapy [14]. The recommended treatment involves systematically reducing DRT while closely monitoring for signs of improvement in impulse control. During this process, it is important to observe for withdrawal symptoms, such as pain, anxiety, irritability, depression, and fatigue. If medication adjustments alone do not suffice, cognitive behavioral therapy (CBT) specifically targeting impulse control disorders should be considered [14].

For DDS, the approach remains consistent with the aforementioned strategies. According to expert consensus on PD, the main approach for managing both punding and DDS involves discontinuing rescue doses of levodopa and apomorphine, followed by a reduction in overall levodopa dose [5]. A retrospective case-control study by Cilia et al. found that long-term positive outcomes for DDS were associated with effective caregiving, and sustained remission was more likely in patients treated with clozapine, duodenal levodopa infusion, or subthalamic nucleus deep brain stimulation (STN-DBS) [12]. Another longstanding strategy is the fractionation of levodopa, which entails administering smaller doses of levodopa more frequently. This aims to minimize dyskinesia by providing smaller doses while preventing the wearing-off effect through increased medication frequency [15].

The management challenges presented in this case are significant and multifaceted. The initial resistance to medication reduction from both the patient and her husband mirrors the difficulties often encountered in managing DDS, where patients may lack insight into their condition and strongly resist changes to their medication regimen [13]. This resistance, coupled with the patient's ability to obtain and take medications even when not prescribed, highlights the need for a comprehensive approach that goes beyond simple medication adjustments. Central to this approach is the importance of education and reassurance for both patients and caregivers. The role of the caregiver, in this case, the patient's husband, is particularly interesting and worthy of reflection. His initial support of the patient's high medication use, followed by a gradual shift in perspective as he observed the benefits of medication reduction, underscores the importance of caregiver education and involvement in DDS management.

The case demonstrates the slow, careful approach often required in DDS treatment. The gradual reduction of the patient's LED from around 2,308 mg to 998.5 mg over several years illustrates the patience and persistence needed. The patient's hospitalization ultimately provided a crucial opportunity for the necessary readjustment of her medication regimen, marking a significant turning point in her treatment journey.

One aspect of the case that diverges from some recommendations in the literature is the lack of CBT in the management strategy. The NICE guidelines suggest that CBT targeting impulse control disorders may be beneficial when medication adjustments alone are insufficient [14]. It would be interesting to consider how the incorporation of CBT might have impacted the course of this patient's treatment.

The case also raises important questions about the relationship between underestimating the impact of pain and the development of DDS in this patient. The patient's severe osteoarthritis and subsequent knee replacements may have complicated her clinical picture, potentially contributing to her pain and reduced mobility, which she misinterpreted as disease progression. This misinterpretation likely led to an increase in her DRT doses. This situation emphasizes the need for comprehensive pain management strategies in PD patients to prevent the misuse of dopaminergic medications for non-motor symptoms [15].

The case additionally sheds light on the potential risks associated with virtual clinics and telephonic consultations in managing complex conditions like PD. Our patient's medication increases primarily occurred during these remote interactions, where decisions were based largely on subjective reports from the patient and her husband rather than objective clinical assessments. The gradual escalation of her LED to 2,308 mg happened through these virtual consultations, without the benefit of in-person evaluation of her motor symptoms, dyskinesias, or behavioral changes. This situation illustrates the importance of caution when adjusting medications remotely, especially for conditions that can impair a patient's insight into their own symptoms. While virtual clinics offer convenience and increased access to care, this case emphasizes the need for periodic in-person assessments, particularly when managing medications with high abuse potential or in patients at risk for impulse control disorders.

## Conclusions

This case provides valuable insights into the presentation, progression, and management of dopamine dysregulation syndrome. It reinforces key findings from the literature while highlighting the inherent variability and complexity in clinical practice. The case emphasizes the importance of educating patients and caregivers on the condition to improve treatment outcomes. Additionally, it serves as a cautionary example of the potential for iatrogenic harm from well-intentioned interventions, stressing the need to differentiate between disease progression and medication side effects, especially in virtual settings where clinical oversight may be less direct.
